# Risperidone Provides Better Improvement of Sleep Disturbances Than Haloperidol Therapy in Schizophrenia Patients With Cannabis-Positive Urinalysis

**DOI:** 10.3389/fphar.2018.00769

**Published:** 2018-07-18

**Authors:** Peta-Gaye L. Thomas-Brown, Jacqueline S. Martin, Clayton A. Sewell, Wendel D. Abel, Maxine D. Gossell-Williams

**Affiliations:** ^1^Section of Pharmacology and Pharmacy, Department of Basic Medical Sciences, The University of the West Indies, Kingston, Jamaica; ^2^Department of Community Health and Psychiatry, The University of the West Indies, Kingston, Jamaica

**Keywords:** *Cannabis*, risperidone, haloperidol, schizophrenia, actigraphy, sleep

## Abstract

A high percentage of persons with Schizophrenia also uses *Cannabis* and this may potentially alter the therapeutic benefits of the antipsychotic medications prescribed. The aim of this study was to assess the impact of *Cannabis* usage on antipsychotic therapy of sleep disturbances in schizophrenia subjects. Male subjects, ≥18 years, admitted to the University Hospital of the West Indies psychiatric ward between October 2015 and October 2016, and diagnosed with schizophrenia were recruited for the study. Following written informed consent to the study, subjects were prescribed either risperidone monotherapy or haloperidol monotherapy orally for 14 days and classified as *Cannabis* users (CU) or non-users (non-CU), with presence/absence of *Cannabis* metabolite in urine samples. After 1 week of admission, subjects wore the Actiwatch-2 device, to record sleep data for 7 consecutive nights. Inferential statistical analysis involved non-parametric tests, expressed as median and inter-quartile ranges (IQR), with *p*<0.05 considered statistically significant. Fifty subjects were assessed, with a median (IQR) age of 28 (14) years. Majority (30; 60%) were CU, displaying longer sleep latency (SL) than non-CU when receiving haloperidol; but equivalent SL when receiving risperidone. In comparison to non-CU, the CU also displayed longer time in bed, but shorter durations asleep, awoke more frequently during the nights and for longer durations, whether receiving haloperidol or risperidone. This resulted in lower sleep efficiency for the CU (<85%) compared to the non-CU (≥85%). Over the study period, sleep efficiency was significantly improved for non-CU receiving either risperidone (*p* = 0.032) or haloperidol (*p* = 0.010); but was only significantly improved with risperidone for the CU (*p* = 0.045). It is apparent that the presence of *Cannabis* may be impacting the therapeutic benefits of antipsychotic drugs on sleep. In schizophrenia subjects with concomitant *Cannabis* use, risperidone is more beneficial than haloperidol in improving sleep efficiency.

## Introduction

The prognosis of schizophrenia is often worsened with *Cannabis* use (Foti et al., 2010; Manrique-Garcia et al., 2014), mainly through potentiation of the well-established dopamine dysregulations in schizophrenia (Bossong et al., 2009, 2015). High percentages of *Cannabis* users (CU) report that it promotes sleep (Cousens and DiMascio, 1973; Schofield et al., 2006; Schaub et al., 2008; Goonawardena et al., 2012; Walsh et al., 2013); however, polysomnographic sleep assessment show relatively inconsistent sleep-promoting properties of *Cannabis* (Gates et al., 2014, 2016). Pharmacological intervention in schizophrenia requires long-term administration of antipsychotic drugs (Mercolini et al., 2007; Fumagalli et al., 2009; Uchida et al., 2011), which block the dopaminergic system. In early polysomnographic studies, typical antipsychotic drugs, such as haloperidol (Clarenbach et al., 1978; Taylor et al., 1991; Maixner et al., 1998; Dursun et al., 1999), demonstrate sleep-promoting effects and improve sleep maintenance/continuity, mainly through the reduction of sleep latency (SL) and frequency of awakening, prolongation of sleep time and an increase in sleep efficiency in healthy subjects and schizophrenia patients (Taylor et al., 1991; Benson, 2006; Cohrs, 2008; Anderson and Bradley, 2013). In contrast to typical antipsychotics, the atypical agents, including risperidone, have demonstrated greater improvement of sleep efficiency, due to the higher affinity for serotonin 5-HT_2A/2C_ receptors, which are involved in controlling sleep quality (Dursun et al., 1999; Ichikawa et al., 2001; Miller, 2004; Cohrs, 2008; Anderson and Bradley, 2013). Furthermore, schizophrenia patients treated with risperidone display better sleep quantity, sleep quality, and general functioning compared to patients treated with typical antipsychotic drugs (Dursun et al., 1999; Yamashita et al., 2002; Giménez et al., 2007; Apiquian et al., 2008; Wichniak et al., 2011).

It is possible for *Cannabis* use to alter the clinical benefits of antipsychotic drugs (Thomas et al., 2015; Patel et al., 2016; Foglia et al., 2017); however, there is a paucity of information evaluating the impact of *Cannabis* use on the sleep outcomes of schizophrenic patients being treated with antipsychotics. Through actigraphy, this study examined the differences in sleep parameters in schizophrenia patients treated with risperidone vs. haloperidol and the impact of *Cannabis* use. Sleep quality can be estimated through sleep efficiency percentage, which incorporates the ratio of total sleep time and total time in bed; both of which can be altered by the SL, wake after sleep onset (WASO) duration and number of awakening (Shrivastava et al., 2014; Reed and Sacco, 2016; Sathyanarayana et al., 2016).

## Methods and Subjects

Ethical approval was obtained from the University of the West Indies Ethics Committee. Males of at least 18 years of age were recruited from the University Hospital of the West Indies’ psychiatric ward between October 2015 and October 2016, if they met the *Diagnostic and Statistical Manual of Mental Disorders, 5th edition* (DSM-V) criteria for schizophrenia, schizophreniform disorder or brief psychotic disorder, as assessed by trained psychiatrists managing each patient. Psychiatric assessments were conducted by three psychiatrists. Written informed consent was obtained from each subject’s relative/guardian on day 1. After consent, urine samples (5 mL) were collected in sterile containers and screened for the possible use of *Cannabis*, using the SD BIOLINE drug of abuse kit, based on the analysis for 11-nor-Δ^9^-tetrahydrocannabinol-9-Carboxylic acid; the main metabolite of Δ^9^-tetrahydrocannabinol, with a detection limit of 50 ng/mL. Subjects were classified as *Cannabis* users (CU) or non-users (non-CU), with a positive or negative kit result, respectively. Psychiatrists managing subjects were blinded from the *Cannabis* result.

Subjects were hospitalized for the 2-weeks study period, and recruited if administered either risperidone or haloperidol, orally at a dose prescribed by the assigned psychiatrists. Therapy could be flexibly adjusted within the therapeutic range as clinically warranted (risperidone, 6–8 mg/day; haloperidol, 10–20 mg/day). In addition, all enrolled subject received daily administrations of benztropine, 2 mg/day. Subjects were excluded from the study if they were receiving any other concomitant medications. Subjects who presented with diagnoses of other central nervous system disorders, mental retardation, somatic diseases, trauma or brain injury and primary sleep disorders were also excluded. Female subjects were excluded to control for the hormone-dependent confounding differences with *Cannabis* use (Craft et al., 2013; Castelli et al., 2014) and sleep disturbances (Sharkey et al., 2014). Demographic information collected from each subject’s docket included age, ethnicity, marital status, occupational status and educational level. History of psychosis (schizophrenia, schizophreniform disorder, or brief psychotic disorder) and previous substances abused (*Cannabis*, tobacco, alcohol, other), if any, were also recorded upon admission to the psychiatric ward.

After 7 days of antipsychotic therapy, only subjects remaining on monotherapy and confirmed by psychiatrist as displaying mild to moderate symptoms (scores < 53), using the Brief Psychiatric Rating Scale (BPRS) were then given the Actiwatch-2 device (Respironics, Inc., Murrysville, PA, United States) to wear on the non-dominant wrist. This device recorded sleep data in each subject for seven consecutive nights, 8 h each night, from 10:00 P.M. to 6:00 A.M. Computer scoring of actigraphy-recorded sleep parameters were performed using Respironics Actiware software, version 5.70.0. The software default setting of immobile minutes was used as the sleep interval detection algorithm. Immobility was determined if the activity counts (AC) was <4 in a 1 min epoch. The data were analyzed using medium wake thresholds (40 AC), with 10 min of immobility set for sleep onset and sleep end. Sleep parameters were calculated during the sleep/rest period (when AC was <40 AC threshold), which was set three different ways: (1) automatic scoring of major rest intervals by the software; (2) pre-determined bedtimes and wake-up times for each subject; (3) manual setting based on surrounding activity level. With the manual setting, the start and end of the sleep period were set close to the pre-determined times on the ward, providing that the AC were not >500 for 2 min, or >1,000 for 5 min, at the start. End of sleep was set when the AC increased to >0 for 5 min, without an epoch scored as sleep. A subject was likely scored as sleep when immobile for 3 h or more. While each of these parameters may have been manipulated by the user, sleep/wake analysis was performed automatically by the software. The scorer was blinded to *Cannabis* status when manually setting the rest intervals. Sleep parameters measured include SL, frequency of awakening, duration of WASO and total sleep time (TST).

## Data and Statistical Analysis

Sleep efficiency percentage (SE%) is a good overall descriptor of a night’s sleep, as it reflects the percentage of time in bed actually spent sleeping (Shrivastava et al., 2014; Reed and Sacco, 2016; Sathyanarayana et al., 2016). SE% was calculated as the percentage of the ratio TST/Time spent in bed (TIB); with TIB calculated as the sum of SL, TST, and WASO. Additionally, sleep efficiency scores were classified as good-quality sleep (SE% ≥ 85%) or poor-quality sleep (SE% < 85%) (Reed and Sacco, 2016; Sathyanarayana et al., 2016). The power of the study was calculated using SE% for CU and non-users, prescribed either haloperidol or risperidone. The power for SE% was >80%, which was adequate. Daily drug dosages were converted to chlorpromazine equivalent (CPZE) doses (Danivas and Venkatasubramanian, 2013) to facilitate drug dosage comparisons.

Statistical analysis was conducted using the Statistical Package for the Social Sciences (SPSS v.20) for Windows (SPSS, Inc., Chicago, IL, United States), with the level of significance set at *p* < 0.05. Demographic data and continuous variables were evaluated using descriptive statistics and expressed as medians and IQR, frequencies (n) and percentages, as appropriate. Subjects were divided into four groups: CU vs. non-CU receiving haloperidol therapy and CU vs. non-CU receiving risperidone therapy. Inferential statistics involved use of Spearman Rho to determine correlations between *Cannabis* grouping and the actigraphy-measured sleep parameters, Kruskal-Wallis test for comparisons across all four groups, with *post hoc* analysis between groups using Bonferroni corrections, and Friedman test for the change in SE% over the seven nights.

## Results

**Table 1** gives demographic information of the 50 male subjects of Afro-Caribbean descent who completed the study. The sample population consisted of 30 subjects (60%) presenting with first-time psychotic episodes (acute psychosis/drug-naive). The remaining 20 subjects (40%) had undergone outpatient therapy or had previously been treated in an inpatient setting. Duration of illness was the only variable significantly different between the acute vs. relapse subjects [0 (2) vs. 11 (1); *p* = 0.024]. Based on self-declaration, initiation of *Cannabis* use was from a median (IQR) age of 14 (2) years. Majority of subjects (*n* = 30, 60%) used *Cannabis* within one week of admission to the psychiatric ward and were classified as CU, with urinalysis. Eighteen CU subjects received risperidone and 12 received haloperidol. The remaining 20 subjects were grouped as non-CU, with 14 receiving risperidone and 6 receiving haloperidol. CU subjects received significantly higher doses of haloperidol compared to non-CU subjects (*p* = 0.001) and CU subjects receiving risperidone (*p* = 0.001). There was no significant difference in dose between CU and non-CU receiving risperidone. By day seven of therapy, there was no significant difference in the BPRS score of schizophrenia symptoms between CU and non-CU receiving haloperidol [30 (6) vs. 28 (9); *p* = 0.335] or risperidone [30 (9) vs. 30 (10); *p* = 0.722].

**Table 1:** emographic characteristics of study participants.

Demographic data		Total (*N* = 50)
Diagnosis (*n*)		Schizophrenia (37), Schizophreniform disorder (7), brief psychotic disorder (6)
Age, years, Median (IQR)		24 (8)
Marital status (*n*)		Single (47), married (1), common-law (2)
Highest education level (*n*)		Primary (6), secondary (41), tertiary (3)
Occupational status (*n*)		Unemployed (48), employed (2)
History of substance use(self-declaration)		*Cannabis* (35), alcohol (27), tobacco/Cigarettes (42), cocaine (2)
**Day 7 CPZE dose (mg/day), Median (IQR)**		
Haloperidol	CU (*n* = 12)	1,000 (375)^a^
	Non-CU (*n* = 6)	750 (500)
Risperidone	CU (*n* = 18)	300 (25)^b^
	Non-CU (*n* = 14)	300 (0)^b^

*^a^p < 0.05 vs. non-CU receiving haloperidol. ^b^p < 0.05 vs. subjects receiving haloperidol. CPZE, Chlorpromazine Equivalent; CU, Cannabis users; non-CU, non-users of Cannabis.*

According to the rank order by Cohen and Holliday (1996), Spearman Rho analysis showed that for subjects receiving haloperidol*, Cannabis* use was moderately correlated to SL (*r* = 0.432, *p* = 0.017), but showed low correlations with TST (*r* = −0.335, *p* = 0.020), WASO duration (*r* = 0.308, *p* = 0.031) and frequency of awakening (*r* = 0.300, *p* = 0.038). This trend was similar for subjects receiving risperidone, with moderate and negative correlation of *Cannabis* use to TST (*r* = −0.433, *p* = 0.017), moderate and positive correlation to WASO duration (*r* = 0.412, *p* = 0.024) and low and positive correlation to frequency of awakening (*r* = 0.381, *p* = 0.035); but no correlation with SL (*r* = 0.336, *p* = 0.064).

**FIGURE 1:**
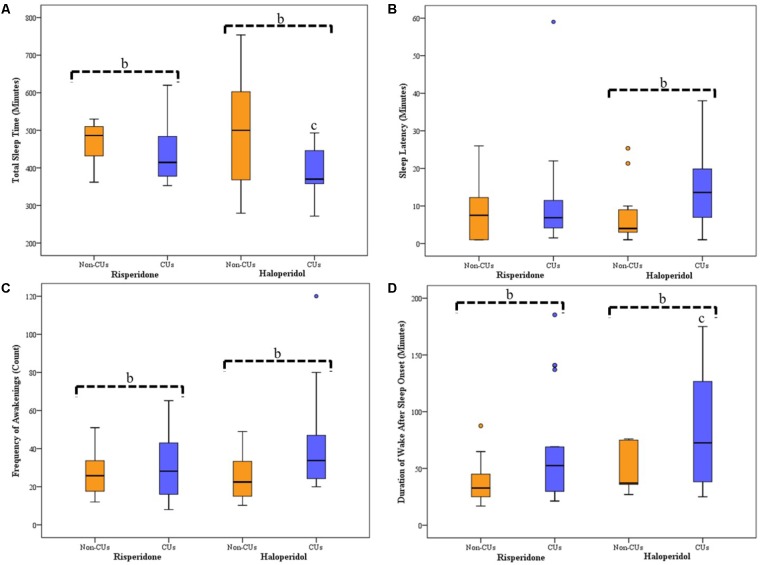
Average Sleep for Seven Consecutive Nights of Actigraphy Recording for CU and Non-CU Receiving either Haloperidol or Risperidone. Box-plots represent median (thick, dark horizontal lines), IQR (boxes), minimum and maximum (whiskers) of each sleep parameter recorded using actigraphy. Circles represents outliers in sleep data. ^b^Significant differences between CU and non-CU groups. ^c^Significant difference between CU treatment groups. **(A)** TST was significantly less for CU than non-CU when receiving either haloperidol (*p* = 0.015) or risperidone (*p* = 0.035); there was significantly less TST between CU receiving haloperidol and CU receiving risperidone (*p* = 0.045). **(B)** SL was significantly longer for CU than non-CU when receiving haloperidol (*p* = 0.009), but not when receiving risperidone. **(C)** Frequency of awakening after sleep onset was significantly more for CU than non-CU when receiving either haloperidol (*p* = 0.017) or risperidone (*p* = 0.004). **(D)** WASO duration was significantly longer for CU than non-CU when receiving either haloperidol (*p* = 0.005) or risperidone (*p* = 0.020); there was significantly longer WASO between CU receiving haloperidol and CU receiving risperidone (*p* = 0.022). CU, *Cannabis* user; non-CU, non-user of *Cannabis*; SL, sleep latency; TST, total sleep time; WASO, wake after sleep onset.

Sleep parameters for CU and non-CU, receiving either haloperidol or risperidone are presented in **Figure 1**. Analysis across the groups showed significant differences for SL (*p* = 0.048), TST (*p* = 0.038), frequency of awakening (*p* = 0.022) and WASO (*p* = 0.030). For the subjects receiving haloperidol, CU subjects displayed significantly longer SL than non-CU subjects [14 (15) vs. 4 (7) minutes; *p* = 0.009] and had significantly less TST [314 (115) vs. 487 (101) minutes; *p* = 0.015]. During sleep, CU subjects had more frequency of awakening [34 (27) vs. 23 (24); *p* = 0.004] and longer WASO durations [73 (94) vs. 37 (42) minutes; *p* = 0.020]. For subjects receiving risperidone, there was no difference in the SL between groups [8 (15) vs. 6 (8) minutes; *p* = 0.088], but CU subjects had significantly less TST [370 (100) vs. 500 (259) minutes; *p* = 0.035], more frequency of awakening [28 (28) vs. 22 (18); *p* = 0.017] and longer WASO durations [53 (75) vs. 34 (21) minutes; *p* = 0.005] than non-CU subjects.

Sleep parameters were not significantly different between the antipsychotic groups for non-CU subjects. Comparisons between CU groups showed no difference in SL and frequency of awakening; however, CU subjects receiving risperidone had longer TST (*p* = 0.045) and shorter WASO duration (*p* = 0.022) than CU subjects receiving haloperidol.

**FIGURE 2:**
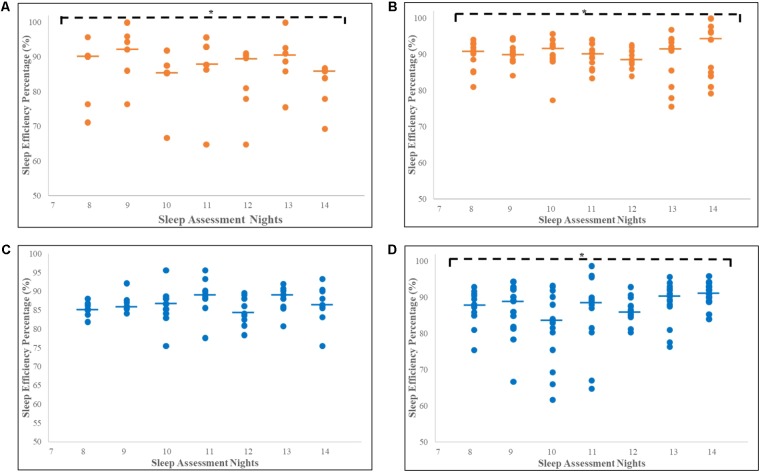
Increase in sleep efficiency percentage for seven consecutive nights for CU and non-CU receiving either haloperidol or risperidone. Each dot represents individual SE% for each subject and the thick horizontal lines indicate the median, on each assessment night. ^∗^Significant increase in SE% over 7 days of recording. **(A)** Significant increase in median SE% for non-CU receiving haloperidol (*n* = 6; χ^2^ = 16.822, *p* = 0.010, *df* = 6). **(B)** Significant increase in median SE% for non-CU receiving risperidone (*n* = 14; χ^2^ = 13.84, *p* = 0.032, *df* = 6). **(C)** No significant increase in median SE% for CU receiving haloperidol (*n* = 12; χ^2^ = 2.78, *p* = 0.180, *df* = 6). **(D)** Significant increase in median SE% for CU receiving risperidone (*n* = 18; χ^2^ = 12.90, *p* = 0.045, *df* = 6). Trend analyses were conducted using Friedman’s tests, with *p* < 0.05 representing significance. CU, *Cannabis* User; non-CU, non-user of *Cannabis*; SE%, Sleep Efficiency Percentage.

SE% for the seven consecutive nights of actigraphy recording for the CU and non-CU receiving either haloperidol or risperidone are presented in **Figure 2**. For non-CU subjects, sleep quality was consistently good (≥85%) over the seven nights and Friedman’s analysis showed significant improvements in SE% when receiving haloperidol (*n* = 6; χ^2^ = 16.82, *p* = 0.010, *df* = 6) or risperidone (*n* = 14; χ^2^ = 13.84, *p* = 0.032, *df* = 6). Good sleep quality and significant improvement in SE% was also recorded for CU subjects receiving risperidone (*n* = 18; χ^2^ = 12.90, *p* = 0.045, *df* = 6). Sleep quality was consistently poor for CU subjects receiving haloperidol and there was no improvement recorded over the 7 days (*n* = 12; χ^2^ = 2.78, *p* = 0.180, *df* = 6).

## Discussions

Risperidone and haloperidol can improve sleep outcomes in schizophrenia patients (Taylor et al., 1991; Wetter et al., 1996; Maixner et al., 1998; Dursun et al., 1999; Haffmans et al., 2001, 2004; Yamashita et al., 2002; Miller, 2004; Monti and Monti, 2004). Similarly, in this study, both risperidone and haloperidol showed improvement in sleep outcomes over the 7 days among schizophrenic subjects not exposed to *Cannabis*. Contrastingly, subjects exposed to *Cannabis* use within 1 week of admission, displayed less total sleep time, with more frequent awakening and longer WASO duration, whether being treated with haloperidol or risperidone; thus, suggesting that the presence of *Cannabis* may be impacting the therapeutic benefits of both drugs on sleep. Converting the antipsychotic doses to CPZEs, our study found haloperidol therapy involved larger doses than risperidone therapy. For subjects exposed to *Cannabis*, the haloperidol dose requirements were much larger when compared with the dose given to non-users. This was not so for risperidone, as the dose was similar between non-users and subjects exposed to *Cannabis*.

Since last use of *Cannabis* by subjects in this study was more than 7 days before actigraphy measurements, the findings may be consistent with signs of *Cannabis* withdrawal on sleep (Bolla et al., 2008, 2010; Babson and Bonn-Miller, 2014; Gates et al., 2014, 2016; Babson et al., 2017). Sleep disturbance is a prominent *Cannabis* withdrawal symptom, usually noted after two nights (Bolla et al., 2008), and progresses over the first 2 weeks (Bolla et al., 2010) of abstinence. Budney et al. (2003) examined the impact of *Cannabis* withdrawal in 18 current users with 12 previous users of *Cannabis* as controls. Users abstained from smoking (confirmed through urine assay for *Cannabis* metabolites) and were assessed using Sleep Inventory questionnaires, administered by telephone daily for 50 days. The study reported sleep difficulty with *Cannabis* use, which peaked 2–6 days after abstinence and remained elevated over a course of 45 days.

Our findings are the first to use actigraphy readings to show that the benefits of haloperidol and risperidone can be significantly impacted by the use of *Cannabis*. Interestingly, risperidone therapy showed better sleep outcomes with recent use of *Cannabis*, as SL was equivalent to non-users. Also, CU awoke for longer durations after sleep onset and thus, slept less, with poor sleep quality when treated with haloperidol. Furthermore, only risperidone was successful at increasing the sleep efficiency among these subjects for the 7 days of observation.

Our findings among the subjects involved in this study may have significant implications, as they suggest risperidone may provide better clinical outcomes on sleep in schizophrenic patients who use *Cannabis;* more so in the Jamaican setting where *Cannabis* use among schizophrenic patients has been established to be high (Knight, 1976; Thomas et al., 2015). However, the positive confirmatory test for *Cannabis* is only able to detect acute *Cannabis* exposure; providing no measure of the history of *Cannabis* use, as documented by self-report of most subjects in this study. Subjects also provided self-report of using alcohol, tobacco and cocaine, which are possible confounders. Despite the presence of these substances and the time of admission not being assessed, the inpatient setting for the study period prevented access. Additionally, some of the subjects in this study previously received therapy with antipsychotics, which could also be a confounder. An antipsychotic-free control group would provide greater comparisons. However, in this setting, this would be unethical since all subjects were experiencing psychosis when admitted to the ward.

## Conclusion

*Cannabis* use can attenuate the benefits of haloperidol and risperidone therapy on sleep in patients being treated for schizophrenia. The better sleep outcomes of risperidone support further examination of its possible superiority over haloperidol. However, the sample size was small, and a larger sample is recommended to confirm findings.

## Ethics Statement

This study was carried out in accordance with the recommendations of the University of the West Indies/University Hospital of the West Indies/Faculty of Medical Sciences Ethics committee guidelines for conducting research. The protocol was approved by the University of the West Indies Ethics Committee. Relatives/Guardians of all subjects gave written informed consent in accordance with the Declaration of Helsinki.

## Author Contributions

The study was designed by MG-W, JM, and WA. P-GT-B reviewed the literature and wrote the protocol for the study. JM, CS, and WA aided in the recruitment of the study participants and performed the psychiatric assessments. Patient recruitment and assessments, sample preparations and assays and the data analyses were conducted by P-GT-B, who also made initial interpretations and wrote the first draft of the manuscript. All authors further interpreted and discussed the findings and contributed to the final version of the manuscript.

## Conflict of Interest Statement

The authors declare that the research was conducted in the absence of any commercial or financial relationships that could be construed as a potential conflict of interest. The reviewer BC and handling Editor declared their shared affiliations.
